# Magnetostrictive Sensor for Blockage Detection in Pipes Subjected to High Temperatures

**DOI:** 10.3390/s19102382

**Published:** 2019-05-24

**Authors:** Alberto M. Pernía, Héctor Andrés Mayor, Miguel J. Prieto, Pedro J. Villegas, Fernando Nuño, Juan A. Martín-Ramos

**Affiliations:** Department of Electrical Engineering, University of Oviedo, 33203 Gijón, Asturias, Spain; hector.andres@eu4m.eu (H.A.M.); mike@uniovi.es (M.J.P.); pedroj@uniovi.es (P.J.V.); fnuno@uniovi.es (F.N.); jamartin@uniovi.es (J.A.M.-R.)

**Keywords:** guided waves (GW), magnetostriction, magnetostrictive sensors (MsS), electromagnetic acoustic transducers (EMATs), non-destructive testing (NDT), molten salts, heat transfer fluid (HTF), thermosolar

## Abstract

The use of solar thermal power plants is considered a cost-effective alternative to produce renewable energy. Unlike other energy installations, in this type of plants the transfer and storage of energy has been solved by using molten salts. These salts run between two tanks through the steam generation system that feeds the turbine. Although the use of salts as a heat transfer fluid is considered an adequate solution, they are not without problems. One of them is the formation of blockages in the pipes due to a partial solidification of the salt, which leads to the shutdown of the installation, with the consequent economic losses. Fast location of these blockages in a minimally intrusive way is the objective pursued in this work. The method to achieve this is based on the use of a new magnetostrictive sensor that simplifies previous designs.

## 1. Introduction

Solar thermal power plants [1] are based on the use of solar radiation for the heating of a heat transfer fluid (HTF) in a field of parabolic trough mirrors. The temperatures to which the fluid is subjected fluctuates depending on the type of technology used, reaching values close to 400 °C when medium-operation temperatures are considered.

Once the fluid reaches the right temperature, it is pumped up to the generation system based on a thermodynamic cycle, commonly a water-steam one, in which the steam turbine is located [2,3,4].

Regarding the types of fluid used we can note, among others, water, air, oil, sodium and different types of salts. The latter are currently considered some of the most suitable alternatives for high temperature power plants [5,6,7,8]. These are eutectic compounds such as KNO_3_-NaNO_3_, 54% and 46% by weight, non-flammable and non-toxic, that melt at temperatures of 222 °C and atmospheric pressure. These compounds are also used as a means of storing thermal energy during periods of excess production, for which there is an isolated tank in which the molten salt is maintained at temperatures above 300 °C.

During periods of low radiation, molten salts stored in the high-temperature reservoir can be used to continue with the generation of electric power. The use of materials that have to be maintained at temperatures above their melting point brings as a consequence the possible occurrence of partial or total blockages, due to the stratification of the fluid itself or to the appearance of cold spots.

**Figure 1:**
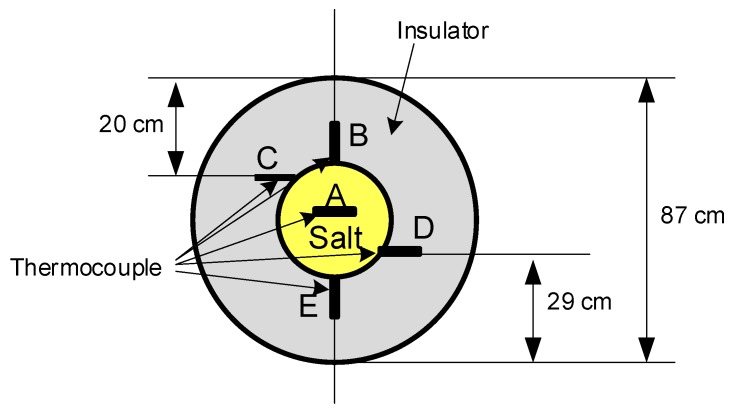
Cross section of a 35-inch diameter pipe with heat transfer fluid.

The temperature control of the pipes that transport the heat transfer fluid requires the incorporation of a resistive heating system (150 W/m^2^ consumption) that, although it has greatly reduced these problems, does not prevent them altogether. It is normal that the plant is forced to maintenance stops due to the formation of plugs in the pipes that carry the molten salt. Direct measurements in real plants show temperature variations of up to 20 °C in a 35-inch diameter pipe for medium-temperature operation. Figure 1 shows the arrangement of thermocouples in different points of the pipeline that allowed the results included in Table 1 to be obtained.

When plants using high-operation temperatures are considered, the maximum temperature in the pipe can exceed 550 °C (Table 2). This increase of the HTF temperature introduces some benefits. As well as improving the efficiency, high working temperatures also reduce the amount of salt used per MWh stored; e.g., working at 550 °C instead of 400 °C reduces the amount of salts required by 40%. Solar thermal power plants that use salt storage temperatures above 550 °C include CRTF (Sandia-New Mexico, NM, USA), Solar Two (Mojave Desert, USA), Tonopah Solar Plant (Las Vegas, NV, USA) or Archimede (Priolo Gargallo, Sicily, Italy) among others.

Location of salt plugs is an arduous task that involves controlling the temperature sensors arranged at fixed points of the pipeline, sectioning portions of the pipe and waiting long times until the temperature of the fluid allows the technical maintenance staff to start operation.

This work focuses on the search for a non-intrusive method based on the use of magnetostrictive sensors that facilitate the precise location of the blockage for its subsequent gradual heating until the salts are melted [9]. This paper is the continuation of previous studies [10] where the signal/noise ratio at different frequencies was analyzed in case of trapezoidal or triangular sensor excitation.

## 2. Magnetostrictive Sensors

The use of waves produced by vibrations in an elastic medium for the analysis of various structures is a widely disseminated non-destructive technique (NDT). The agitation of the molecules, caused by the disturbance, will propagate as a deformation wave in the medium, where it is observed that the propagation speeds of the different types of waves depend exclusively on the material and are independent of the frequency and amplitude of the excitation. When moving, the wave is affected by the discontinuities of the medium, which will cause reflections. From the information provided by these reflections, it will be possible to deduce the presence of the defect and the distance from the origin of the disturbance.

The types of waves that occur in the medium are classified according to the direction of propagation of the wave front in relation to the direction of the displacement of the particles. Hence, it is possible to have longitudinal, transverse, surface or torsional waves. Torsional waves propagate in cylindrical bodies in the form of bars or tubes. They are rotational oscillations of the molecules of the solid around the longitudinal axis of the solid that coincides with the direction of propagation. This is the type of wave this work is focused on. The torsional mode is considered optimal due to its non-dispersive characteristics [11]. There are extensive studies on the use of this technique, introduced for the first time by Rose [12,13]. Torsional waves can be generated by using the magnetostrictive effect that gives rise to mechanical stress in certain ferromagnetic materials, such as nickel, when subjected to a magnetic field. The inverse effect also takes place: application of mechanical stress in these materials can generate a magnetic field. Therefore, the magnetostrictive effect can be used both for the generation of the torsional wave and for its detection [14].

Despite the existence of piezoelectric transducers, which could also be used for the goal pursued, their continuous use at high temperature (200 °C) is discarded (note that commercial piezoelectric transducers normally have a Curie temperature around 350 °C, lower than the maximum temperatures that can be present in the pipes considered). That is why this work focuses on magnetostrictive devices, which are especially interesting when dealing with extreme temperatures [15,16,17,18].

### 2.1. Magnetostrictive Transducer

**Figure 2:**
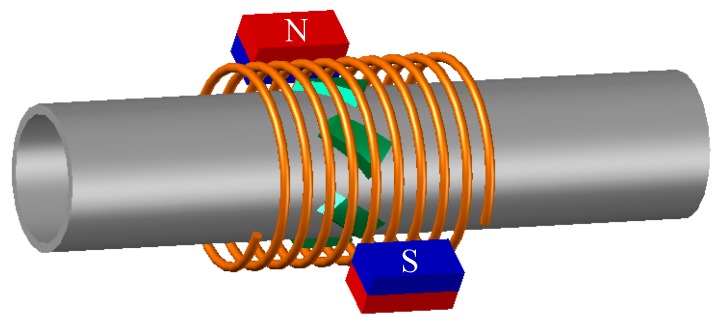
Design of the magnetostrictive transducer.

The solution proposed by Kwun [19] for the construction of a magnetostrictive transducer is based on the use of the magnetostrictive properties of nickel. Several strips of this material are attached to the pipe and then surrounded with a solenoid that is used to create an alternating magnetic field. Additionally, two magnets are placed in the vicinity to cause the premagnetization of the nickel strips (Figure 2).

**Figure 3:**
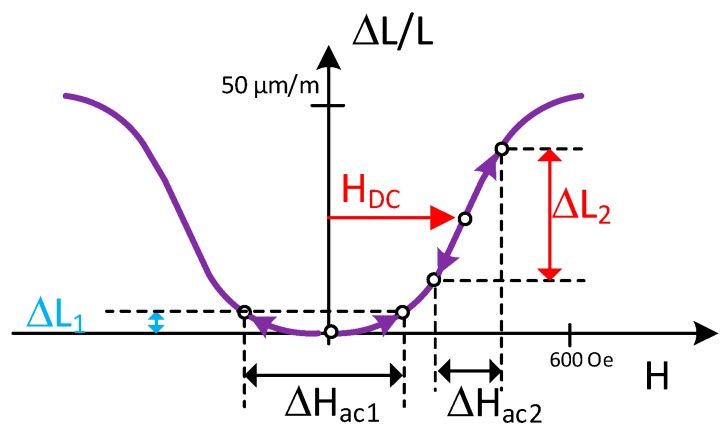
Magnetostrictive behavior.

Premagnetization of the nickel strips improves the amplitude of the response of the transducer and its linearity with lower values of the alternating field. Figure 3 shows the variation in the material elongation depending on whether a continuous magnetic field H_DC_ is applied or not [20].

The composition of the continuous field introduced by the magnets together with the alternating field generated by the solenoid results in a deformation in the nickel strip that is transmitted in the form of a torsional wave in the aluminum tube. Such a system has already been developed and there is commercial equipment [21,22,23,24,25] used to detect defects and discontinuities, where the use of solenoids that completely surround the pipeline is considered in all cases.

Two problems can be identified in this structure. On the one hand, the sensor needs a large area of the thermal insulator material to be removed in order to place the AC coil and the magnets all around the pipe. On the other hand, even if high-temperature magnets are used (the temperature range of SmCo magnet alloys goes up 350 °C, e.g., VACOMAX 145/170/200, and AlNiCo magnets can actually be used up to 530 °C without irreversible losses in magnetizing, e.g., LN10 from Hangzhou Permanent Magnet Group (Hangzhou, China), the maximum operation temperature of these devices is too close to the HTF temperature.

Therefore, a design is proposed in this paper that aims to adapt the use of magnetostrictive transducers for the detection of blockages in pipes, trying to avoid the temperature limitation introduced by magnets (even considering those applications where HTF temperature can be well above 550 °C). Also, the design presented can be introduced through the thermal insulating material by just drilling a 5-cm-diameter hole.

**Figure 4:**
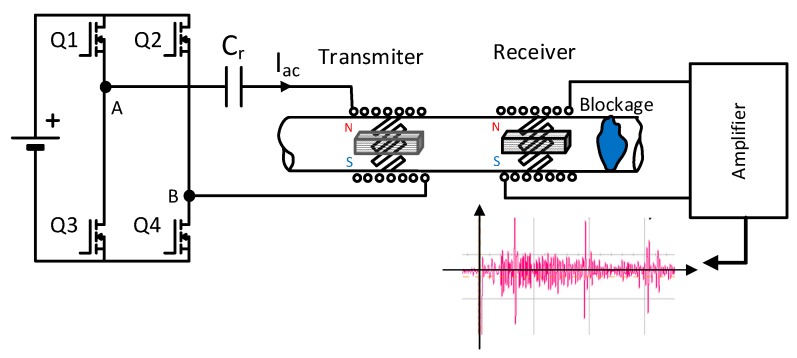
Test bench.

In order to do so, a preliminary test bench is used (Figure 4) where the generator of the torsional wave (transmitter) has been implemented with 8 strips of permendur (cobalt-iron alloy 49Fe-49Co-2 V) arranged at 45°, surrounded by a solenoid on which two permanent NdFeB magnets are placed, as indicated in Figure 2 [26,27,28,29]. Permendur has been selected due to the greater elongation (∆L/L) that it presents as compared with other types of magnetostrictive materials (Table 3). A dry coupling with the pipe is carried out despite the response penalization this type of coupling gives rise to; this is so because the ultimate goal is to design the system for high-temperature applications in which metal strips will be used to fix the magnetostriction material [30,31].

The receiver responsible for detecting the torsional waves reflected by the blockage is based on the same technology. A full bridge converter is used to inject a sinusoidal signal in the transmitter by means of a resonant topology whose resonant elements are a resonant capacitor C_r_ and the inductance of the external winding.

**Figure 5:**
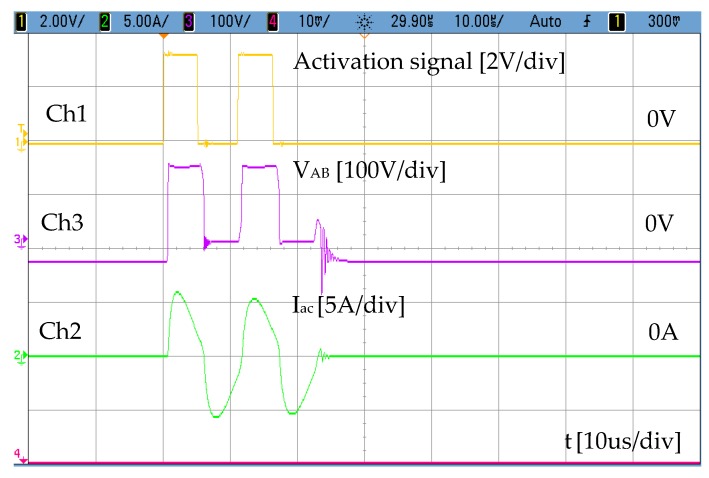
Activation pulse of the magnetostrictive transducer. Ch1: Digital signal to the power stage, Ch2: current through the AC coil, Ch3: Voltage at the resonant circuit.

As indicated above, the power supply used with the transmitter is a bridge converter in which a resonant capacitor C_r_ has been included in order to produce a current through the solenoid as sinusoidal as possible. The amplitude of current I_ac_ is fixed by the appropriate selection of the input voltage, whereas the adequate selection of the resonant capacitor, C_r_, is a function of the operating frequency. By activating transistors Q1 and Q4, the first positive pulse between terminals A-B is generated V_AB_ = 150 V during 5 µs. After this, Q1 is turned off and Q3 is turned on to define a dead time of 5 µs where V_AB_ = 0. The process is repeated to generate the next positive pulse. Transistor Q2 is only used for synchronous rectification purposes. Figure 5 shows the current injected into the solenoid (Ch2 ≈ 5A_peak_) together with the output voltage of the inverter bridge. (Ch3). The voltage generated between terminals A-B (Ch3) responds to the activation signal generated with a Texas Instruments TMS320F28335 microcontroller (Ch1). As mentioned above, transformation of voltage V_AB_ into a sinusoidal current is achieved by means of a resonant circuit that consists of a capacitor, C_r_, and the winding coil used in the transmitter. The value C_r_ = 94 nF is selected to operate at 100 kHz while keeping the input voltage of the power stage lower than the rectified AC grid. As it can be seen in Figure 5, the excitation current obtained through the transmitter winding (Ch2) is quite sinusoidal.

**Figure 6:**
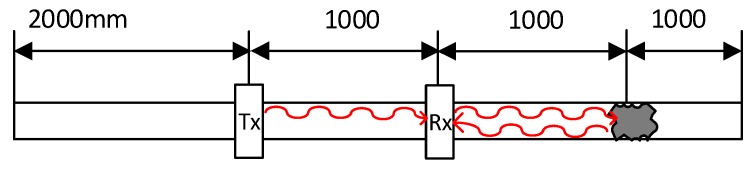
Location of the transmitter, receiver and blocking.

**Figure 7:**
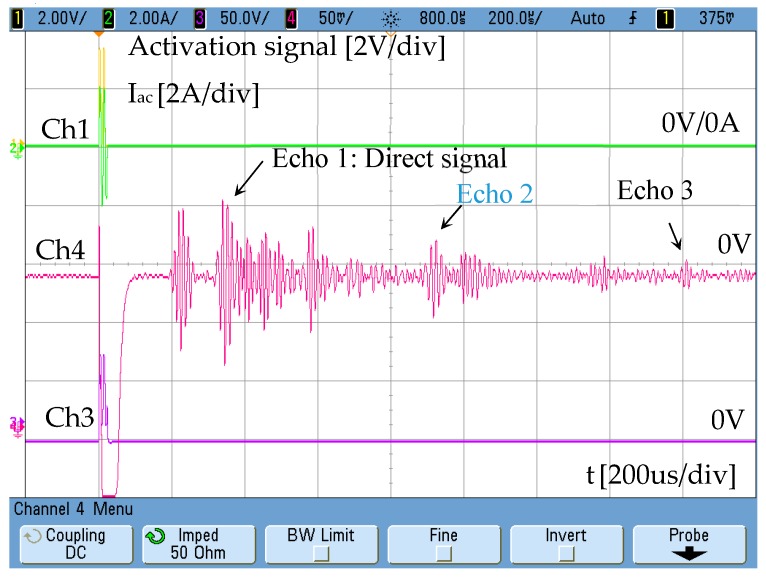
Magnetostrictive transducer response Ch4: 50 mV/div.

The current injected in the external winding consists of two sinusoidal cycles with a low content of high-frequency harmonics. In the test performed, a tube blockage made with cement has been placed 2 metres away from the emitter (Figure 6). The aluminium pipe has Ø37 mm inner diameter and is 3-mm thick. The response obtained by the receiver is shown in Figure 7. The speed of the wave along the aluminium tube is 3120 m/s.

The distance between receiver and emitter is 1 m, so the signal corresponding to the detection of the blockage matches echo 2. Echo 1 corresponds with the direct signal between transmitter and receiver. Echo 3 is related to the reflection of the wave on pipe ends.

### 2.2. Characterization of the Magnetostrictive Transducer

If modifications are to be included in the transducer so as to facilitate its use in high-temperature environments, it is necessary to identify the fields that are being handled by the device. From finite-element simulations, the field distribution in the permendur strips can be determined when an alternating current of 5 A_peak_ is applied in a solenoid formed by 50 turns.

**Figure 8:**
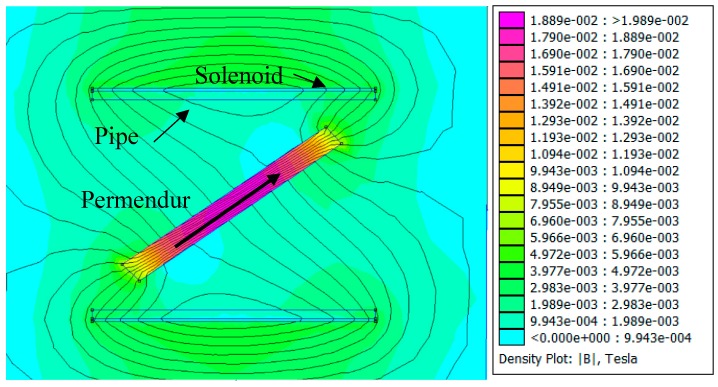
Activation pulse of the magnetostrictive transducer.

Figure 8 shows that the solenoid used tends to generate a magnetic field whose direction is parallel to the axis of the pipe and which is forced to penetrate the permendur strip along its longitudinal direction with an amplitude of 20 mT. Thus, although field generation with a solenoid in a direction other than the longitudinal direction of the permendur strip is possible, it is definitely not the most efficient way to achieve such a goal.

**Figure 9:**
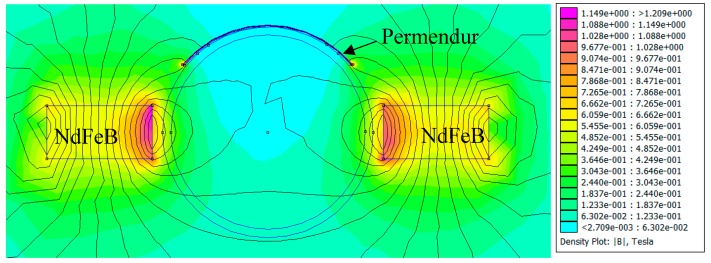
Penetration of the magnetic field created by two permanent NdFeB magnets in the permendur strip. Maximum magnetic field obtained in permendur strip: 1.3 T.

Figure 9 shows the magnetic field created by the permanent magnets that generate the continuous magnetic polarization of the magnetostrictive material in order to increase its response and linearity. It can be seen that very high values are obtained on the surface (>0.5 T), and that high flux-density fields are created along the longitudinal direction of the permendur strip.

**Figure 10:**
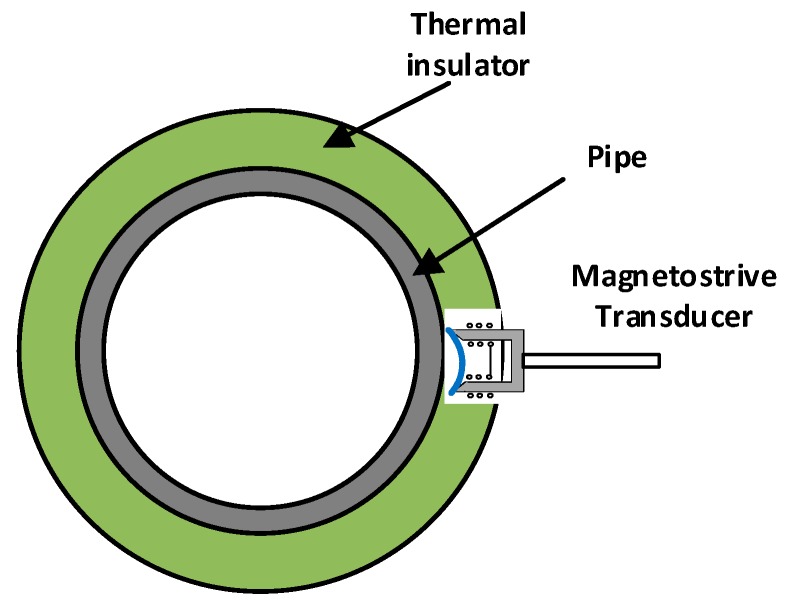
Positioning of the transducer.

These simulations show that both the continuous and the alternate magnetic field follow the longitudinal direction of the permendur strip, which evinces that it is possible to apply both using structures that do not need to be wound around the pipe. This would facilitate the placement of the transducer and make it possible to design a device similar to a temperature probe, which would be introduced all the way through the thermal insulator to the pipe (Figure 10).

Thus, a new design is proposed that winds the solenoid around a magnetic core that will be placed on the permendur strip. Additionally, the DC polarization is carried out by adding another winding around the aforementioned magnetic core through which a direct current will be injected.

**Figure 11:**
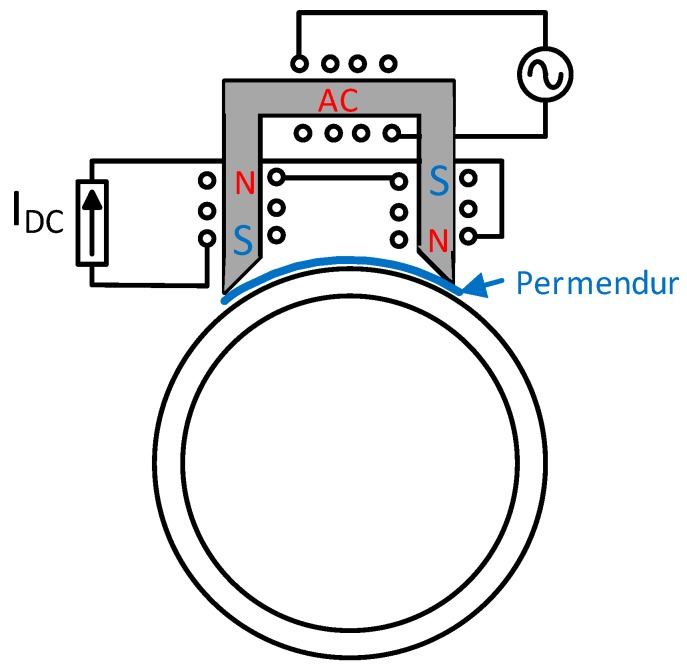
New magnetostrictive transducer.

By doing so, the permanent magnets have been replaced by a DC winding and the alternating magnetic field is now applied directly through the permendur strip using the AC winding, both with the direction of the longitudinal section of the strip (Figure 11). 

**Figure 12:**
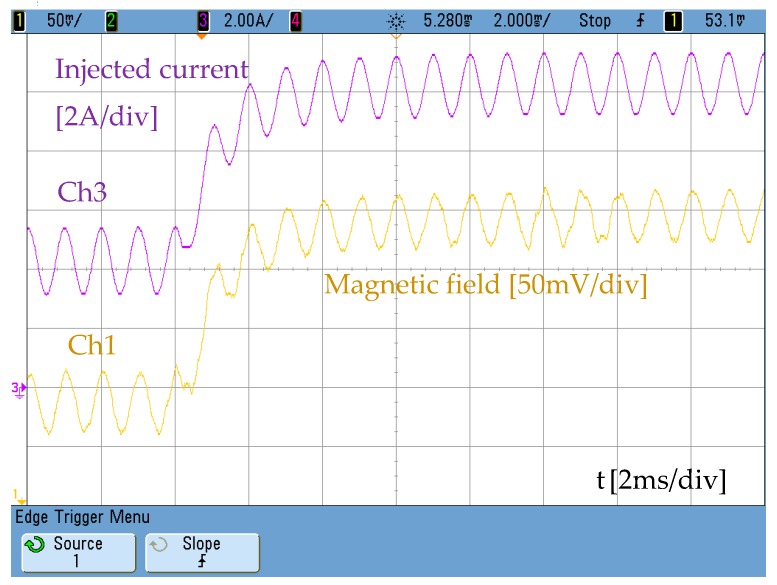
Measurement of the magnetic field generated when a DC current step is applied. Trace 1-yellow (12 V/Tesla), trace 3-purple: current through the AC winding.

The effect of both windings will produce an alternating magnetic field with a DC polarization that can be externally controlled. Figure 12 shows the alternating magnetic field with a DC component created by mixing both DC and AC currents.

**Figure 13:**
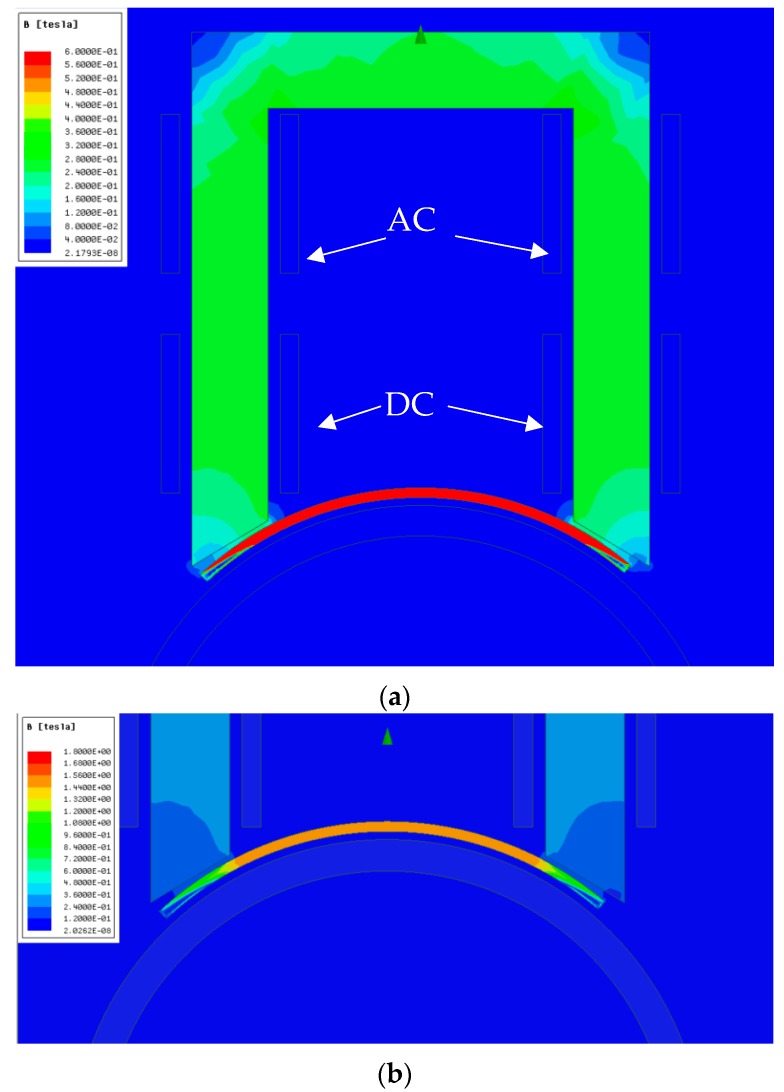
Magnetostrictive transducer based on the use of a magnetic core. Simulation performed at 0 Hz and 5 A. Maximum magnetic field in the core (**a**) and permendur (**b**).

The simulation of Figure 13 shows the performance of the alternative described above. Two windings are arranged around a magnetic core of rolled steel, the first of which is meant to create a continuous magnetic field below saturation (DC in Figure 13a) that allows the working point in the curve of Figure 3 to be defined as a function of the injected direct current. The upper limit of the legend in Figure 13a is too low to read the actual magnetic field in the permendur strip. This limit has been increased in Figure 13b so as to see it clearly.

**Figure 14:**
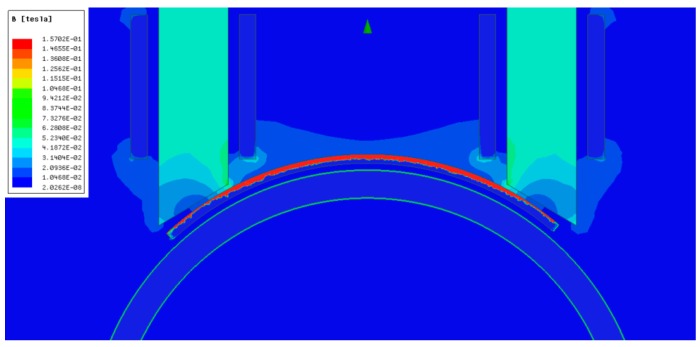
Magnetostrictive transducer based on the use of a magnetic core. Simulation performed at 100 kHz, 5 A.

The other winding (AC in Figure 13a) is connected to the full bridge and its mission is making the permendur sheet vibrate, thus producing the torsional wave in the pipe. Since the magnetic core is close to the permendur sheet, the field is perfectly confined inside the sheet (Figure 14). In all the simulations carried out, the maximum current used is 5 A_peak_.

The signal generated by the full-bridge on the AC winding (Figure 4) will have a frequency around 100 kHz so as to obtain quasi-sinusoidal currents through the AC coil with an amplitude of 5 A when the components selected are used. Increasing the operating frequency will result in the effective surface of the permendur layer being reduced as shown in Figure 14, where only the upper part of the strip has a significative magnetic field. Therefore, it would be advisable to select the strip thickness close to twice the skin depth (*δ*) in order to improve the magnetic field penetration (*δ* = 0.03 mm for permendur at 100 kHz).

(1) $$\delta =\sqrt{\frac{\rho }{\pi \mu f}}\;\left[m\right]$$

where *ρ* is the conductivity, *µ* is the permeability and *f* is the operation frequency.

**Figure 15:**
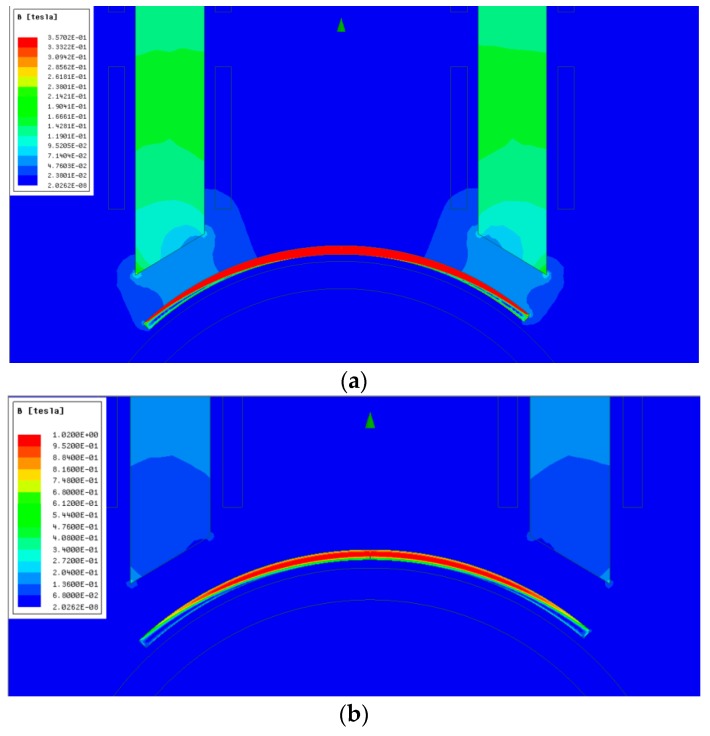
Magnetic field distribution by moving the magnetic core 3 mm (**a**). Detail of the magnetic field inside the permendur strip (**b**).

Even when the core is displaced 3 mm from the surface of the permendur strip (Figure 15), a good concentration of the field on the surface of the magnetostrictive material continues. The increased distance, however, makes the amplitude of the magnetic field be reduced to ≈30% the value obtained when the core is placed closer (see Figure 13) and the same current through the windings is used, 5 A in this case. 

## 3. Results

**Figure 16:**
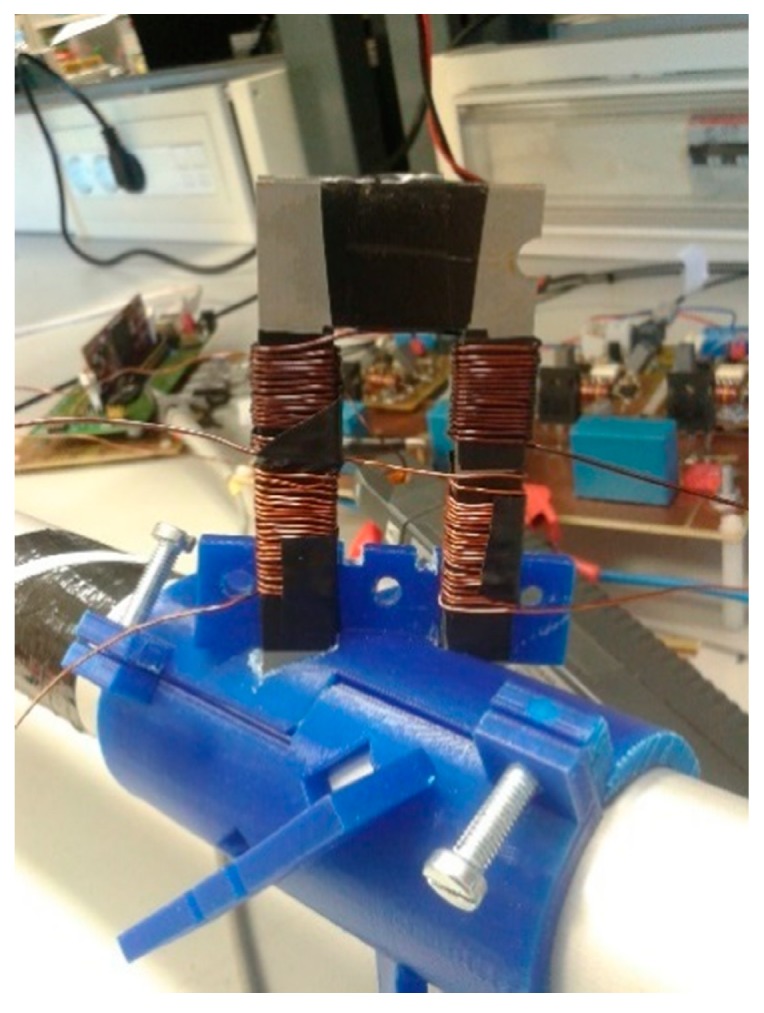
Magnetostrictive transducer based on the use of a magnetic core.

The transducer defined in the previous section has been constructed to demonstrate its viability. Two 50-turn windings have been defined: one of them will be used for the AC pulses and the other one will handle a DC current for the DC magnetic polarization. Figure 16 shows the magnetic material used (oriented grain) and its placement on an aluminum pipe 40 mm in diameter.

**Figure 17:**
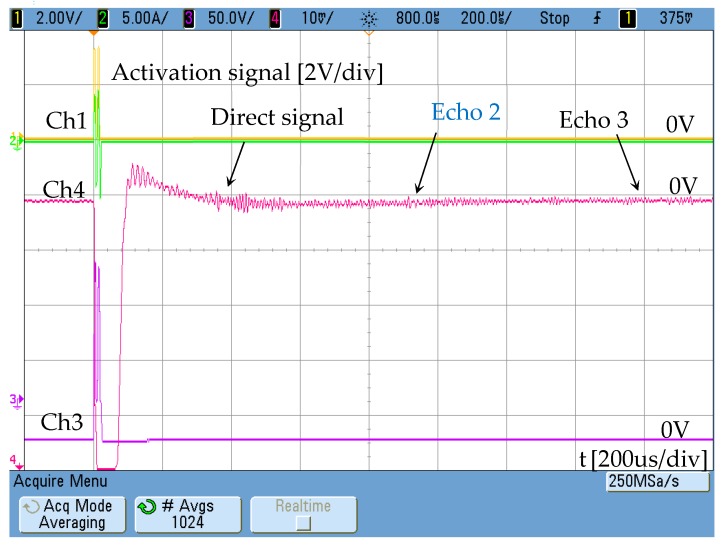
Magnetostrictive transducer response without DC bias, Ch4: [10 mV/div]. Ch1 shows the activation signal of the power stage [2 V/div] and Ch3 the voltage at resonant circuit [50 V/div]. The maximum AC current is 5 A.

The transducer consists of only one permendur strip, unlike the one previously used, where eight strips were arranged around the aluminium pipe (Figure 2). The strip dimensions are: 50 × 5 × 0.25 mm. In the mentioned conditions, and using the transducer indicated in Figure 2 as a receiver, the transmitter is initially supplied from the full bridge without injecting any current through the DC winding and removing the permanent magnets. Therefore, the permendur strip is not magnetically polarized. Using a blockage made with solid cement, the result obtained is an extremely weak signal in which it is difficult to identify the echoes produced (Figure 17).

**Figure 18:**
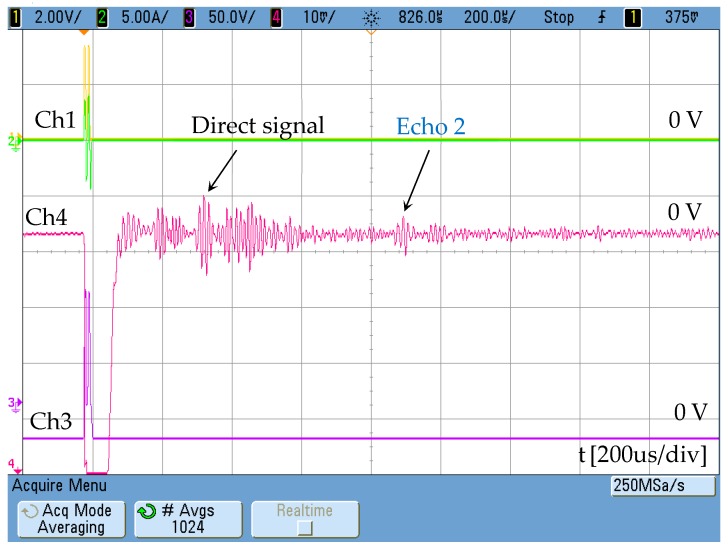
Magnetostrictive transducer response with DC magnetic bias (two NdFeB magnets) Ch4: [10 mV/div]. Ch1 shows the activation signal of the power stage [2 V/div] and Ch3 the voltage at resonant circuit [50 V/div]. The maximum AC current is 5A.

The effect of the magnetic polarization in the permendur strip can be clearly detected if two magnets are placed following the schematic in Figure 2. In this case the signal obtained (Figure 18) allows echoes to be detected at the same intervals measured with the traditional electromagnetic transducer (Figure 2).

Subsequently, the permanent magnets were removed, and a DC 5-amp current was applied through the winding arranged for this purpose in order to check the effectiveness of the DC winding.

**Figure 19:**
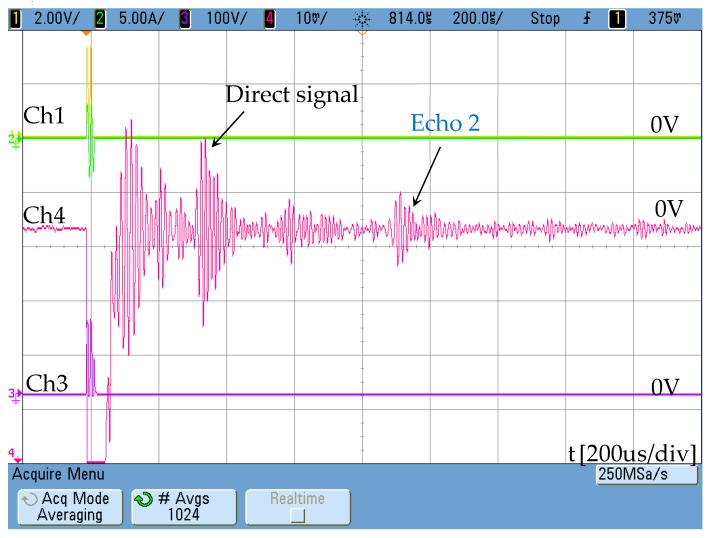
Magnetostrictive transducer response with DC current bias. Ch4: [10 mV/div]. Ch1 shows the activation signal of the power stage [2 V/div] and Ch3 the voltage at resonant circuit [100 V/div]. The maximum AC current is 5 A.

A notable improvement in the response of the sensor is observed (Figure 19), also introducing the possibility of controlling the constant magnetic field with the DC current applied. Although the signal remains weak, it must be taken into account that it has only been generated using a single strip of permendur.

Another characteristic to keep in mind is the possibility of using the transducer proposed as a receiver. If this is feasible, the positioning of the receiver and transducer is greatly simplified, allowing its use through the thermal insulation of the pipe.

**Figure 20:**
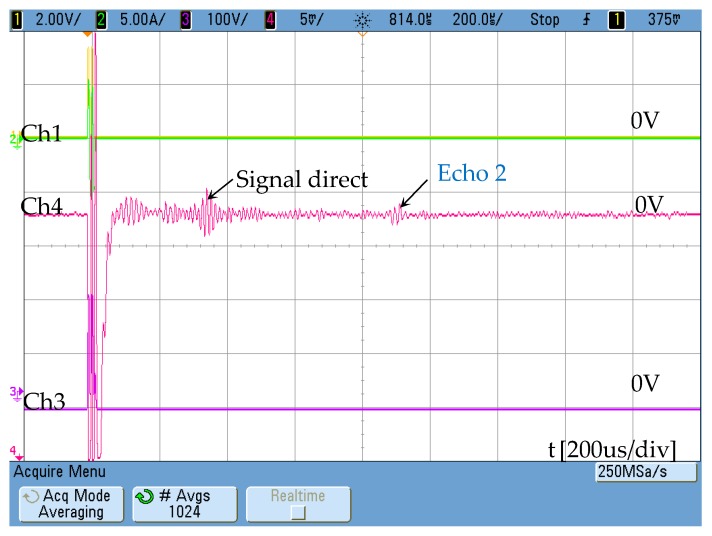
Signal obtained in the receiver configured according to the new magnetostrictive transducer using permanent magnets (Figure 10) Ch4: [5 mV/div]. Ch1 shows the activation signal of the power stage [2 V/div] and Ch3 the voltage at resonant circuit [100 V/div]. The maximum AC current is 5A.

In order to check its operation as receiver, the same configuration indicated in Figure 11 has replaced the traditional receiver but using permanent magnets instead of the DC coil. The response obtained (Figure 20) is again lower than that obtained with the receiver based on Figure 2, but its reception capacity is demonstrated.

**Figure 21:**
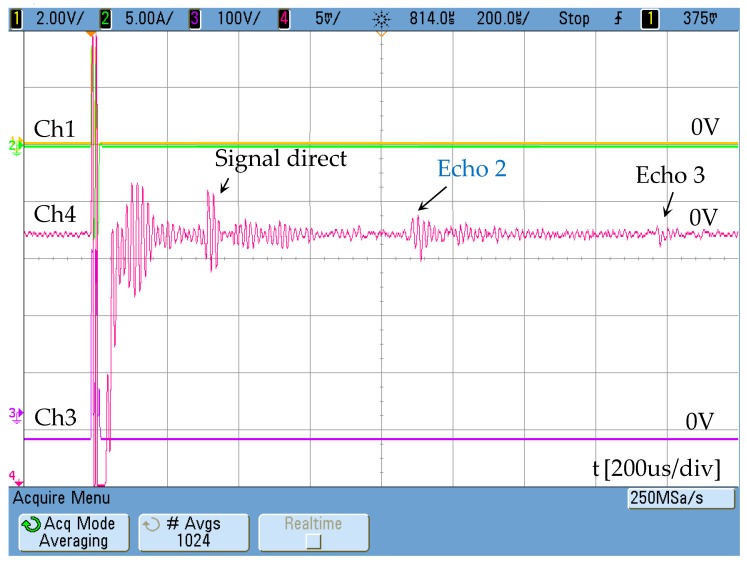
Signal obtained in the receiver configured according to the new magnetostrictive transducer with pre-magnetized strip Ch4: [5 mV/div]. Ch1 shows the activation signal of the power stage [2 V/div] and Ch3 the voltage at resonant circuit [100 V/div]. The maximum AC current is 5 A.

With the aim of eliminating any permanent magnet a new test was carried out using permendur strips previously premagnetized. This was done by applying a 10-amp constant current through the DC coil before operating the receiver. The results obtained confirm the detecting capability of the proposed transducer (Figure 21).

It must be noticed though that, due to the transducer low signal response obtained, simultaneous operation of the power supply to inject a constant current through the DC coil during the signal reception increases the noise background, making it difficult to identify the blockage echo. In order to avoid this noise introduced during the measurement by the power supply, it will be used initially to create the DC polarization by means of the DC coil and then turned off while performing the actual measurement using the AC coil (together with the DC polarization still present in the material).

All the signals in the previous figures have been obtained directly from the oscilloscope (MSQ6104A-1 GHz, Agilent, Santa Clara, CA, USA) using only an averaging filter.

The signal obtained can be improved by using digital filters that allow the signal to be processed and reduce the complexity of the circuitry. With this aim, the wavelet (WT) transform is used to increase the signal-noise ratio. The wavelet decomposition is based on a set of functions ψ(*t*) called mother wavelet:

(2) $$\psi_{\tau ,s}=\frac{1}{\sqrt{s}}\cdot \psi \left(\frac{t-\tau }{s}\right)$$

where “*t*” is the translation and “*s*” is the scale.

The wavelet transformation is then defined as:

(3) $$WT_{\tau ,s}=\int_{-\infty }^{\infty }f\left(t\right)\psi_{\tau ,s}^{*}\left(t\right)\mathrm{dt}$$

To check the filtering capability of WT, the signal obtained in Figure 21 was transformed by using Wavelet Discrete Meyer (dmey) level 3. The parameter d_3_ obtained provides a better precision to detect the echos.

**Figure 22:**
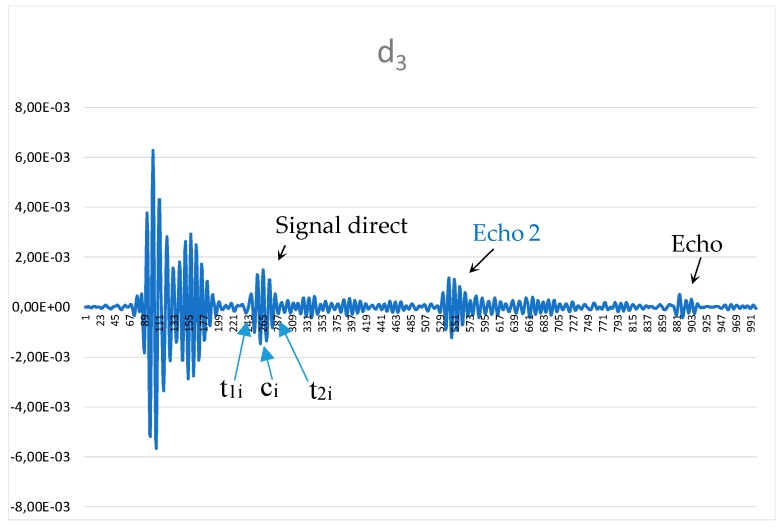
Signal obtained from the wavelet transformation [V/div].

**Figure 23:**
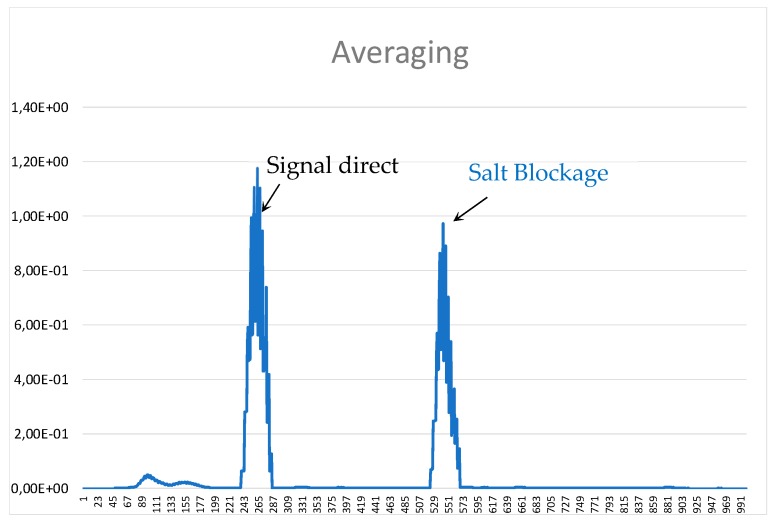
Signal obtained from the wavelet transformation post-processing.

Once the signal has been filtered (Figure 22), a post-processor can be applied to determine the position of the salt blockage. Something as simple as defining a reference threshold to eliminate amplitude signals below that value and then identifying the start (t_1i_), centre (c_i_) and finish (t_2i_) of the peaks whose amplitude is higher than the threshold. After this, the absolute average value of this piece of signal is assigned to the centre of each peak burst multiplied by the desired gain produces information as clear as that shown in Figure 23.

Other signal processing techniques [32,33] can be used for higher localization resolution. In this application, however, high accuracy is not essential, given the notable size of the blockage. What is extremely important is to develop a sensor that can be used in real thermal pipes avoiding the removal of the thermal isolation.

## 4. Discussion

Modification of electromagnetic transducers in high-temperature applications involves adapting their geometry. In solar thermal power plants, the detection of blockages in pipes through which the heat transfer fluid (molten salts) flows presents several problems to solve. One of them is reducing the elimination of the thermal insulation layer that surrounds the pipe. The use of transducers that require the entire pipeline to be surrounded by a coil involves removing a significant part of the mentioned thermal insulation. The proposal made in this work aims to develop a transducer that only needs thermal insulation to be drilled away in a very specific area. The price to pay is a lower-amplitude response as compared with the signals obtained when typical transducers (like the one shown in Figure 2) are used.

The use of materials that can withstand high temperatures is another problem that limits the design of electromagnetic transducers. Piezoelectric devices are discarded because their operating temperature is relatively low, around 150 °C. Therefore, metallic materials are suggested instead, such as permendur for the magnetic strips (with a Curie temperature of 940 °C) and grain-oriented silicon iron for the core of the transducer (the Curie temperature of which is 740 °C). In both cases the saturation induction is higher than 1 T, which is also interesting to increase the magnetic field in the magnetic core if the gap should be increased. Additionally, the performance of the proposed sensor is not to be limited by the maximum operation temperature of the magnets included in other versions of this type of sensors. This is achieved by replacing these magnets with a simple DC coil where all materials used can easily operate above 700 °C.

## 5. Conclusions

The use of a manetostrictive sensors based on the structure proposed by Park for detection of blockages in pipes is proposed. Starting from the traditional structure, a modification of the sensor is proposed to facilitate its use in high-temperature systems, which are usually protected by a thick insulating coating. The proposed system consists of a single device composed of a magnetic core that polarizes a strip of permendur, which remains in contact with the pipe to generate a torsional wave. Not only does the proposed sensor allow the torsional wave to be generated, it can also be used as a receiver of such a wave. The design of the sensor was accomplished by running several finite element simulations with Maxwell-Ansys program to identify the magnetic field evolution.

The proposed design defines a practical configuration that can be used at high temperature as a thermal probe would, without the need to remove all the thermal insulation. Research challenges that can be identified include improving the response of the sensor and optimizing the frequency and intensity of the currents, both AC and DC.

## Figures and Tables

**Table 1:** Measurement of the temperature in the solar power plant “La Africana”.

Thermocouple	Salt Temperature (°C)	External Temperature (°C)
TT-13WSX30CT001-JTC1 position A	312,25	
Thermocouple position B	294	286
Thermocouple position C	290	282
Thermocouple position D	285	277
Thermocouple position E	281	273

**Table 2:** Heat transfer fluid (HTF) temperatures.

HTF	Minimum Temperature (°C)	Maximum Temperature (°C)
Nitrate salts	265	565
Sodium liquid	270	530
Carbonate salts	450	700

**Table 3:** Longitudinal magnetostriction of several elements and alloys.

Material	∆L/L
Nickel	−33 × 10^−6^
Cobalt	60 × 10^−6^
45 Permalloy	27 × 10^−6^
Permendur-2 V	70 × 10^−6^
